# Cell type-resolved human lung lipidome reveals cellular cooperation in lung function

**DOI:** 10.1038/s41598-018-31640-x

**Published:** 2018-09-07

**Authors:** Jennifer E. Kyle, Geremy Clair, Gautam Bandyopadhyay, Ravi S. Misra, Erika M. Zink, Kent J. Bloodsworth, Anil K. Shukla, Yina Du, Jacquelyn Lillis, Jason R. Myers, John Ashton, Timothy Bushnell, Matthew Cochran, Gail Deutsch, Erin S. Baker, James P. Carson, Thomas J. Mariani, Yan Xu, Jeffrey A. Whitsett, Gloria Pryhuber, Charles Ansong

**Affiliations:** 1 Biological Sciences Division, Pacific Northwest National Laboratory, Richland, WA 99352 USA; 2 Department of Pediatrics, University of Rochester School of Medicine and Dentistry, 601 Elmwood Avenue, Rochester, NY 14642 USA; 3 Division of Pulmonary Biology, Cincinnati Children’s Hospital Medical Center, Cincinnati, OH 45229 USA; 4 Genomics Research Center, University of Rochester School of Medicine and Dentistry, 601 Elmwood Avenue, Rochester, NY 14642 USA; 5 Flow Cytometry Core Facility, University of Rochester School of Medicine and Dentistry, 601 Elmwood Avenue, Rochester, NY 14642 USA; 6 Texas Advanced Computing Center, University of Texas at Austin, Austin, TX 78712 USA; 7 Department of Pathology, Seattle Children’s Hospital, Seattle, WA 98105 USA

## Abstract

Cell type-resolved proteome analyses of the brain, heart and liver have been reported, however a similar effort on the lipidome is currently lacking. Here we applied liquid chromatography-tandem mass spectrometry to characterize the lipidome of major lung cell types isolated from human donors, representing the first lipidome map of any organ. We coupled this with cell type-resolved proteomics of the same samples (available at Lungmap.net). Complementary proteomics analyses substantiated the functional identity of the isolated cells. Lipidomics analyses showed significant variations in the lipidome across major human lung cell types, with differences most evident at the subclass and intra-subclass (i.e. total carbon length of the fatty acid chains) level. Further, lipidomic signatures revealed an overarching posture of high cellular cooperation within the human lung to support critical functions. Our complementary cell type-resolved lipid and protein datasets serve as a rich resource for analyses of human lung function.

## Introduction

Cell type-resolved organ maps hold significant promise in facilitating/providing a deeper understanding of human organ functions. Recent cell type-resolved transcript and/or proteome analyses of the heart, brain and liver have been reported generating heart, brain and liver transcriptome and/or proteome maps. Sharma *et al*.^1^ applied high resolution mass spectrometry-based proteomics and deep sequencing transcriptomics to characterize four major cell types in the mouse brain. Utilizing a similar proteome-transcriptome strategy Azimifar *et al*.^2^ characterized five hepatic cell types in the mouse liver. More recently Doll *et al*.^3^ described a cell-type resolved proteomic map of the human heart analyzing three major cardiac cell types. While cell type-resolved organ transcriptome and proteome maps are now becoming available, similar organ maps for the lipidome are needed.

The lung is a complex organ comprised of multiple cell types each playing overlapping and niche roles in facilitating normal lung development and function. Molecular profiling, including proteomics, lipidomics and transcriptomics, of mouse tissues is increasingly used to understand lung morphogenesis and function^4–8^. While these earlier efforts have been useful they provide an averaged view of a biological system. There is increasing ability to use sorted and single cell data to identify crucial information on niche and cooperative actions/activities of specific cell populations. Recently, a cell type-resolved transcriptome analysis of murine lung epithelial cells^9^ and a human alveolar epithelial type 2 (AT2) cell transcriptome at single cell levels have been described^10–13^. Lipids mediate important biochemical functions of the lung and, not being directly encoded by nucleic acids, are not necessarily correlated or adequately inferred from mRNA abundance. Important in the lung in particular, lipids are the majority (90%) component of pulmonary surfactant, a lipid-protein complex that decreases alveolar surface tension preventing atelectasis during the respiratory cycle^14^. Additionally lipids have essential roles in lung development and repair, intra- and inter-cellular signaling and mediation of inflammation^15,16^. Lipidomic analyses of isolated mouse alveolar type 2 cells have been reported^17–19^, however other major lung cell types have not been characterized; either in mouse or humans. Thus characterization of the lipidome of individual human lung cell types will help to better understand the processes regulating normal lung formation and function.

Liquid chromatography-tandem mass spectrometry (LC-MS/MS)-based lipidomics provides a powerful means of examining lipidomic signatures of biological samples in an unbiased manner. In this study we used LC-MS/MS-based analyses to define the features of major lipids in specific cellular compartments of the lung from three 20 month old donors (D01, D08 and D11). Four major lung cell types were isolated by fluorescence-activated cell sorting techniques and the functional identity of the isolated cells substantiated via LC-MS/MS-based proteomics. Subsequent LC-MS/MS-based lipidomic profiling of the four lung cell types highlighted unique lipid profiles across cell types and revealed highly interconnected and coordinated cellular networks within the human lung that support critical lung functions of gas exchange and the innate host response. Our cell type-resolved lipid data, complemented by cell-type resolved transcript and protein data, serve as a rich resource for study of lung development and function.

## Results and Discussion

### Sorted cell populations are substantiated by protein profiling

**Figure 1:**
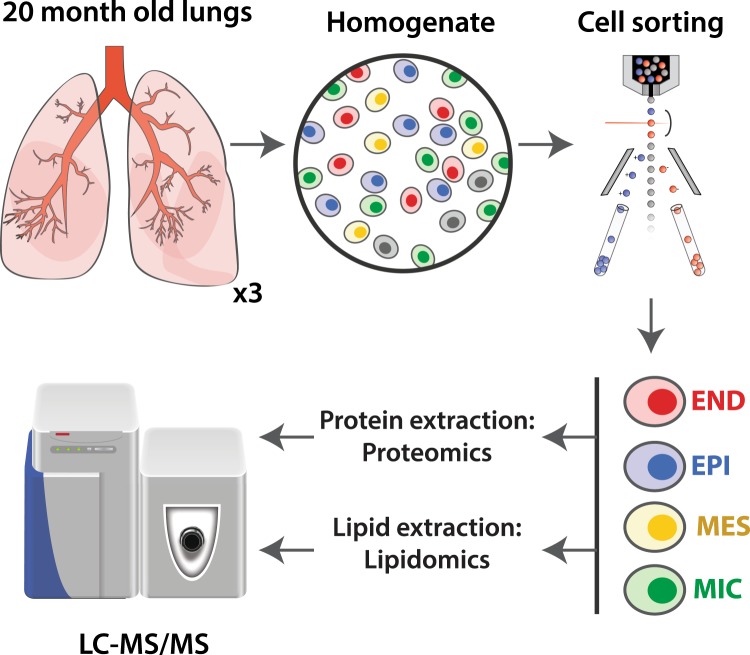
Lipidomic and proteomic analysis of lung cell types. Schematic workflow for the cell-type-resolved lipidomics and proteomics of human lung.

**Figure 2:**
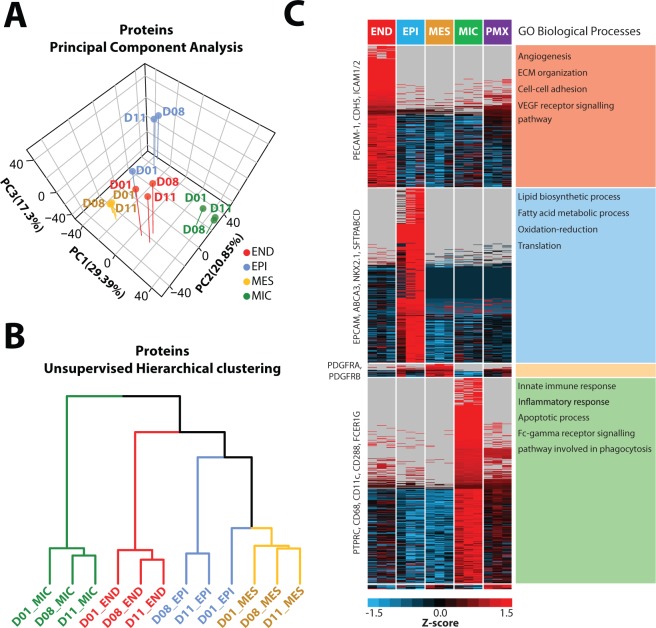
Principle component analysis (**A**) and hierarchical clustering (**B**) of cell type-resolved proteomics data from three donors (D01, D08, D11). (**C**) Heatmap of proteins differential across four cell types isolated from donors. Data shows a high degree of similarity between cell types for all three donors. Known markers indicated within cell-specific clusters. Annotation enrichment analysis highlights physiological role of cells.

Lung samples were collected from three 20-month old female donors (Table S1). To facilitate a cell-type-resolved analysis of the lung lipidome, four major cell types, endothelial (END), epithelial (EPI), mesenchymal (MES) and mixed immune (MIC), cells, were isolated from each donor lung (Fig. 1). Cell types were positively selected on the following CD markers: CD31/144+ for END, CD326+ for EPI, CD45+ for MIC cells. Mesenchymal (MES) were the remaining cells isolated after negative selection. The four cell types were isolated via FACS as described in Methods. To substantiate the functional identity of isolated cells, the four isolated cell populations were subjected to global protein profiling. Data from the protein profiling (Table S2) of the four cell types are freely available at Lungmap.net and the LGEA web portal (https://research.cchmc.org/pbge/lunggens/mainportal.html). Principal component analysis of the proteome data partitioned the four cell types into well-defined populations (Fig. 2A). Cluster analysis of proteins that were differentially expressed across the four cell populations were identified by ANOVA (p < 0.05) revealing preferential enrichment of subsets of proteins including well-known markers specific for each cell type (Fig. 2B). Functional annotation analysis of these subsets utilizing DAVID^20^ revealed top-ranking GO BP terms associated with each cell type (Fig. 2C, Tables S3) and supported the known physiological roles of the isolated cells, validating the fidelity of the FACS sorting process.

### LC-MS lipidome profiling of major types of lung cells

**Figure 3:**
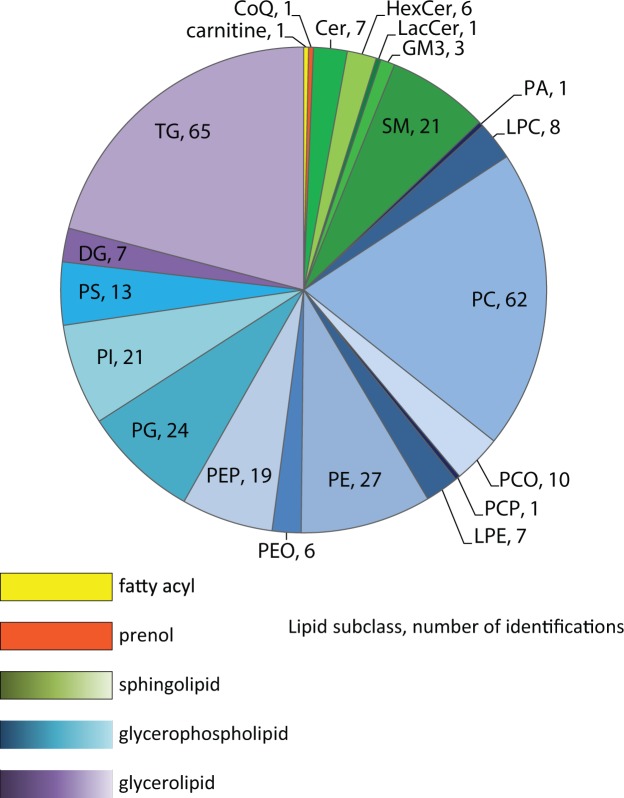
Distribution of lipids identified in cell-type resolved human lung. Total of 311 unique lipids identified across 5 lipid categories, including fatty acyls (yellow), prenol (orange), sphingolipids (greens), glycerophospholipid (blues), and glycerolipid (purples), and 21 subclasses. CoQ10 = coenzyme Q10; Cer = ceramide; HexCer = glucosyl- or galactosylceramide; LacCer = lactosylceramide; GM3 = ganglioside; SM = sphingomyelin; PA = diacylglycerophosphate; LPC = monoacylglycerophosphocholine; PC = diacylglycerophosphocholine, PCO = ether PC; PCP = plasmalogen PC; LPE monoacylglycero-phosphoethanolamine; PE = diacylglycerophosphoethanolmine; PEO = ether PE; PEP = plasmalogen PE; PG = diacylglycerophosphoglycerol OR bis(monoacylglycerol)phosphate; PI = diacylglycerophosphoinositol; PS = diacylglycerophosphoserine; DG = diacylglyceride; TG = triacylglyceride. Values beside each subclass annotation represents the number of lipids identified in that particular subclass.

**Figure 4:**
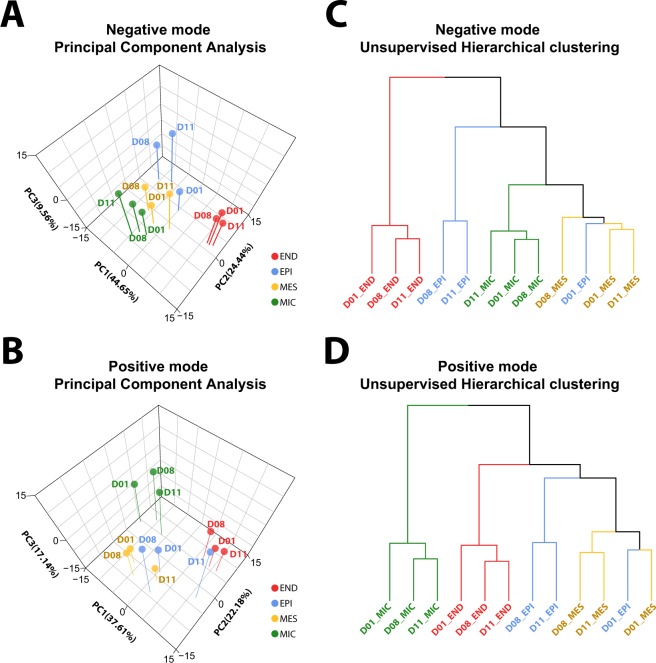
Molecular similarity within cell types. Principle component analysis (PCA) and hierarchical clustering (HC) of cell type-resolved lipidomics data collected in negative (**A**) and positive (**B**) ionization modes from three donors (D01, D08, D11). Data shows a high degree of similarity within the cell types with the exception of D01 EPI, which appears more similar to the MES cells in the lipidome.

The lipidome of the four major cell types from each of the three donors, as well as an unsorted, mixed cell population, used as a control, was profiled using an LC-MS/MS platform previously described^6,21^. We employed LIQUID^21^ for lipid identification and quantification. We identified 311 unique lipids across 5 lipid categories (e.g., sphingolipid, glycerophospholipid, and glycerolipid) and 21 subclasses (e.g., ceramide (Cer), diacylglycerophosphocholine (PC), and triacylglyceride (TG)) in each of the cell types (Fig. 3; Table S4). PCA and hierarchical clustering (HC) analysis of the lipidomics data partitioned the samples into well-defined cell-specific clusters (Fig. 4). For sorted END, MES and MIC cells, cells from each of the three donors (D01, D08 and D11) clustered well within a specific cell type: EPI cells from D08 and D11 clustered well together, while EPI cells from D01 were more similar to MES cells (Fig. 4). PCA and HC analysis of proteomics data generated from the same samples supported this observation (Fig. 2A,B). Interestingly, the pathology review for donor D01 (Table S1) indicated acute bronchopneumonia that may have influenced the results.

### Lipid subclass and intra-subclass vary among four major lung cell types

**Figure 5:**
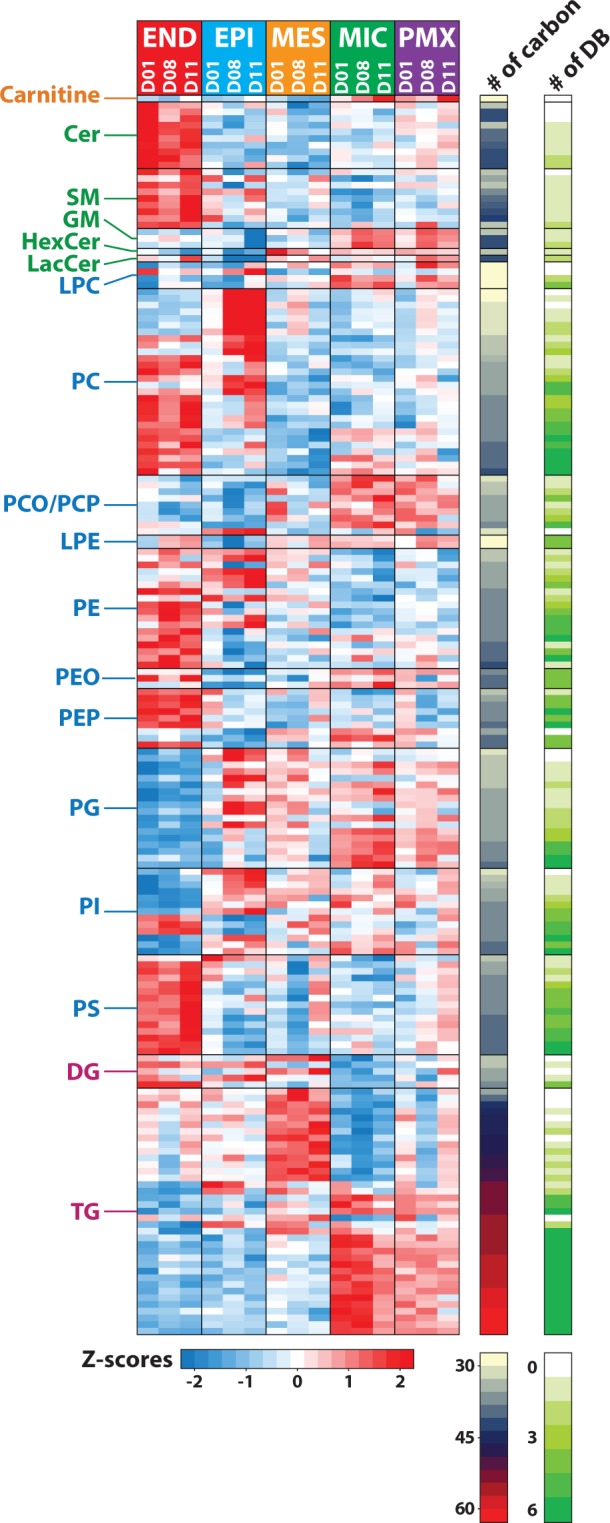
Lipid subclass and intra-subclass profiles across the four cell types. Heatmap visualization of statistically significant (p-values < 0.05) lipidome of sorted END (endothelial), EPI (epithelial), MES (mesenchymal), and immune (MIC) cells and unsorted control cells (PMX) for the three donor (D01, D08, D11) human lung samples. Data in the heatmap is z-scored and sorted at the subclass level based on the total hydrocarbon chain length and then the total of double bonds in hydrocarbon chains. CoQ10 = coenzyme Q10; Cer = ceramide, SM = sphingomyelin; GM3 = ganglioside; HexCer = glucosyl- or galactosylceramide; LacCer = lactosylceramide; PA = diacylglycerophosphate; LPC = monoacylglycerophosphocholine; PC = diacylglycerophosphocholine, PCOP = ether (O) and plasmalogen (P) PC; LPE monoacylglycerophosphoethanolamine; PE = diacylglycerophosphoethanolmine; PEO = ether PE; PEP = plasmalogen PE; PG = diacylglycerophosphoglycerol OR bis(monoacylglycerol)phosphate; PI = diacylglycerophosphoinositol; PS = diacylglycerophosphoserine; DG = diacylglyceride; TG = triacylglyceride.

**Figure 6:**
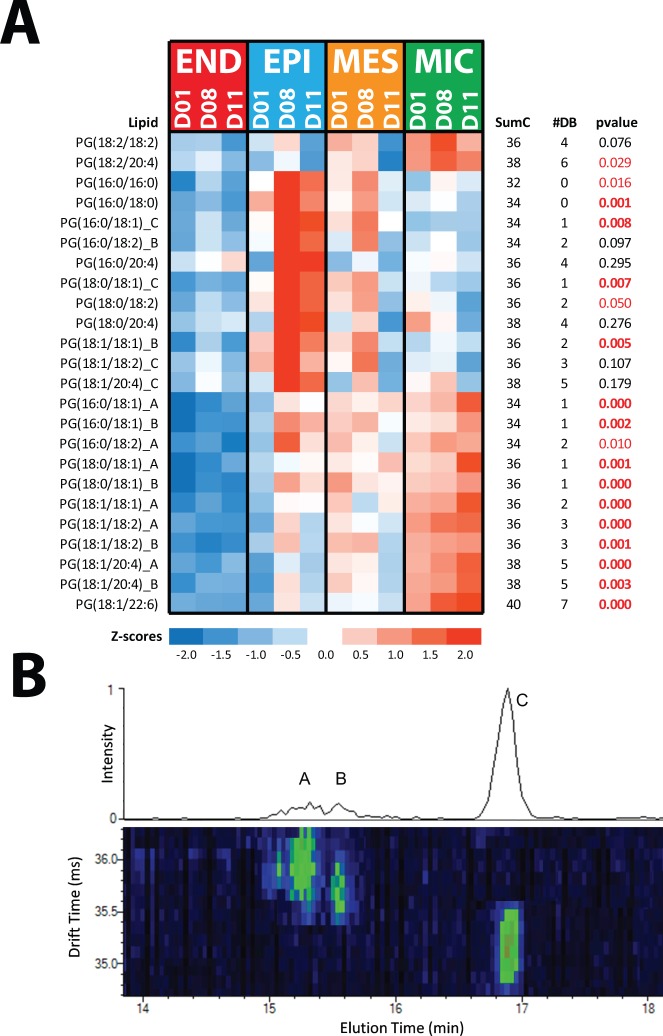
LC-IMS-MS distinguishes PG from bis(monoacylglycerol)phosphate (BMP). (**A**) Heatmap of identified PG lipids. Data in the heatmap is z-scored. SumC represents the total number of carbons in the fatty acids chains, and #DB represents the total number of double bonds in the fatty acids chains. The p-values highlighted red are statistically significant (≤0.05) and in red bold text for those with p-values ≤ 0.01. (**B**) Representative LC_IMS-MS analysis of EPI shows three isomers of PG(16:0_18:1) noted as A, B, and C. In the IMS analyses, the structural sizes were found to be in the order of A>B>C, where A was only slightly bigger than B, but both were quite a bit larger than C (see drift time separation). Previously observed BMP were found to be larger than PG (Kyle *et al*.^22^), illustrating that A and B are likely BMP isomers. In the LC separation, A and B eluted very close together and ~1.5 minutes earlier than C also fitting the LC elution time differences between BMP and PG. A PG(16:0_18:1) standard was evaluated and found to match the elution time of C. Taken together, the above observations indicate that A and B, the isomers enriched in MIC cells, are BMP isomers, and identifies the main isomer in EPI cells, C, as PG(16:0_18:1) the primary PG surfactant lipid.

Quantification of the identified lipids revealed that 187 species varied in a statistically significant manner (t-test; p < 0.05) in cross-comparisons of each cell-type to the other and the unsorted control (PMX) cell population (Table S5; Table S6 and Fig. 5). MIC lipids were the least variant relative to the control PMX cells with only 17 lipids (5.6%) being significantly different (p < 0.05). END lipids were most variant relative to control PMX cells with 40% of the lipids being different (p < 0.05) (Table S6). At the lipid category level, sphingolipids were enriched in END cells, glycerophospholipids enriched in EPI cells and glycerolipids enriched in MES and MIC cells (Table S5). Lipid profiles among the four cell types varied most at the subclass and intra-subclass (total carbon length of the fatty acid chains) levels (Fig. 6; TableS5). Inter-subclass differences were identified for diacylphosphoglycerols (PG). PG isomers with shorter LC retention times (RT) (e.g., PG(16:0_18:1)_A, PG(16:0_18:1)_B) were increased in MIC cells, whereas PG isomer(s) with longer LC RTs (e.g., PG(16:0_18:1)_C) were most abundant in EPI cells (Fig. 6A; Table S5). The difference in PG isomer enrichment in EPI and MIC was not related to intact total fatty acid number of carbons or double bonds, indicating a core structural difference. A total of 52 stereoisomers were identified in this study comprising 24 isomer groups (Table S4); however, only the PG isomers differed in a cell-type specific pattern, suggesting a functional role for these particular PG structural isomers.

### Isomer characterization supports presence of bis(monoacylglycerol)phosphate (BMP) lipids in human lung immune cells

Structural isomer characterization of intact lipids (e.g., sn position, orientation and position of double bonds along the fatty acid chains) is difficult utilizing traditional LC-MS. Liquid chromatography coupled with ion mobility spectrometry and MS (LC-IMS-MS) has recently emerged as a way of characterizing lipid structural isomers^22^. LC-IMS-MS analyses were conducted on the EPI and MIC cells to elucidate the structural differences between PG isomers exhibiting cell-type specific patterns (Fig. 6B). Specifically examining three PG isomers PG(16:0_18:1)_A, PG(16:0_18:1)_B, and PG(16:0_18:1)_C (Fig. 6B) LC-IMS-MS structural characterization identified the ‘C’ isomer enriched in the EPI sample as PG(16:0_18:1), the primary PG surfactant lipid, while the ‘A’ and ‘B’ isomers enriched in the MIC samples were identified as bis(monoacylglycerol)phosphate (BMP) isomers.

BMP lipids are highly enriched in the inner membrane of late endosomes^23,24^ and lysosomes acting as biomarkers for these organelles. These organelles play important roles in autophagy and phagocytosis. The presence of these lipids in MIC supports active autophagy and/or phagocytosis processes. Protein pathway enrichment data support this insight (Table S3). Proteomics identified lysosomal acid lipase (LICH_Human), a protein associated with lysosomes, as statistically significantly enriched in MIC (Figure S1; Table S2). BMPs are enriched in rodent alveolar macrophages and phagosomes but not in polymorphonuclear leukocytes (or granulocytes)^25^. Our present data support the presence of BMP in human lung immune cells.

### Characterization of mesenchyme cells support the presence of lipofibroblasts

**Figure 7:**
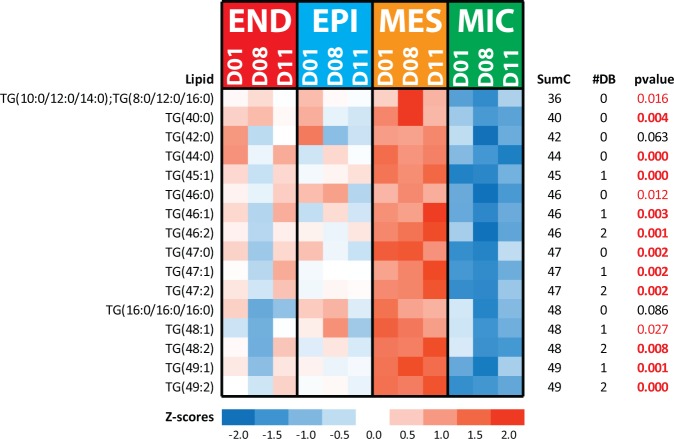
Heatmap of TG lipids elevated in MES cells. Data in the heatmap is z-scored. SumC represents the total number of carbons in the fatty acids chains, and #DB represents the total number of double bonds in the fatty acids chains. The p-values highlighted red are statistically significant (p-value ≤ 0.05) and in red bold text for those with p-values ≤ 0.01. Note the elevated TG lipids have low sumC and #DB in the MES when compared to MIC cells (Fig. 8).

MES cells, located at alveolar septal tips^26^, are important cells that direct lung development^27,28^ and have multiple functions including driving epithelial branching and differentiation, and alveolar maturation^28^. MES cells serve as progenitors of lipofibroblasts, which are required for alveologenesis in murine models (McCully *et al*. 2012). MES-derived lipofibroblasts store TGs, and TGs within lipofibroblasts are located in close proximity to alveolar type II cells^27^ and act as a source of fatty acids for surfactant production within alveolar type II cells^29,30^. The presence of lipofibroblasts in human lung has been a subject of debate^31,32^. Lipidomic profiling of MES cells in the present study revealed shorter chained (C36-49) TG were the only group of lipids enriched in MES cells (Fig. 7; Table S5) and were dominated by fully saturated (8 out of 10 TGs with no double bonds) and monounsaturated TGs (5 out of 8 TGs with a total of 1 double bond) TGs. All of the TGs increased in MES cells contain all or a combination of 14:0, 16:0, 16:1, and 18:1 fatty acids, which are the main fatty acids in surfactant lipids (e.g., PC(16:0/16:0), PC(16:0_16:1), PC(16:0_18:1), PC(14:0_16:0), and PG(16:0_18:1))^33^. The observed saturation state of the fatty acids could account for some of the debate regarding the presence of lipofibroblasts in human lungs as the staining techniques employed by^32^ for TEM visualization of lipids in assessing the presence of lipofibroblasts in human lung require unsaturated fatty acids to react with osmium tetraoxide^34^. In addition to the lipidomics analysis, transcriptomic data^10^ (Figure S2) supported increased TG hydrolysis and the presence of lipid droplets in MES. Lipoprotein lipase (LPL), which releases fatty acids from TGs^35^, was highly expressed in MES compared to other cell types (Figure S2). Perilipin-2 (PLIN2), a known protein marker associated with lipid droplets, was also highly expressed in MES (Figure S2). Taken together the present lipidomics data, supported by transcriptomic and proteomic data, support the concept that lipofibroblasts are present in human lungs similar to rodent/murine lungs.

### Alveolar epithelial cells and surfactant production

Alveolar type II epithelial cells are well appreciated as a major source for surfactant lipids secreted into the alveoli. Surfactant lipids comprise ~90% of pulmonary surfactant (surfactant proteins comprise the remaining ~10%), a lipoprotein complex that functions to decrease surface tension in the post-natal lung and prevent lung collapse. PCs represent the major lipid component of pulmonary surfactant (~60%), followed by PGs (7–15%)^33,36^. Lipidomics analysis of isolated EPI cells in the present study revealed increased abundance of short chained PC (fatty acid (FA) carbon length C28–34) lipid species (Figure S3; Table S5). Among these were well known surfactant lipid species such as POPC (PC(16:0_18:1); FA carbon length C34) and DPPC (PC(16:0/16:0); FA carbon length C32). Proteomics data showed that surfactant proteins SP-A, B, C, D were enriched in EPI cells (Table S2; Figure S4) consistent with the primary role of alveolar epithelial cells in surfactant production. DPPC, a saturated PC is the most abundant lipid in pulmonary surfactant, representing ~40% of total surfactant mass^14^. DPPC is produced from *de novo* synthesis of PC via the Kennedy pathway and remodeling of unsaturated PC species via the Lands cycle. Proteomics data revealed that LPCAT1, an enzyme critical in the Lands cycle production of DPPC^14,37^, was most abundant in EPI cells (Figure S5A) while a key enzyme in the Kennedy pathway, the rate controlling PCY1A (Asgassandian 2013),^14^, was not enriched (Figure S5B). In the fetal lung, *de novo* synthesis contributes highly to surfactant production at birth while in the postnatal lung there is already a reservoir of surfactant available and pool sizes are increasingly maintained by recycling. Thus we speculate that where our donors were in mid to later stages of alveolarization, which occurs from 36 weeks preterm to 36 months postnatal in humans^38,39^, DPPC is preferentially produced via the Lands cycle.

### Lipid signaling is a feature of lung immune cells

**Figure 8:**
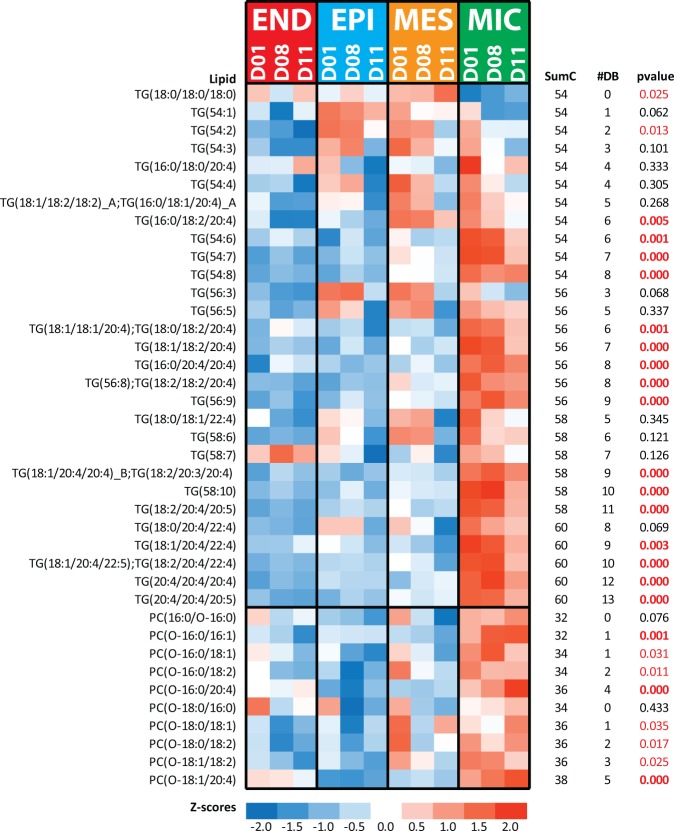
Heatmap of TG and PCO lipids elevated in the MIC cells. Data in the heatmap is z-scored. SumC represents the total number of carbons in the fatty acids chains, and #DB represents the total number of double bonds in the fatty acids chains. The p-values highlighted red are statistically significant (p-value ≤ 0.05) and in red bold text for those with p-values ≤ 0.01. Note the elevated TG lipids have higher sumC and #DB in the MIC when compared to the MES (Fig. 7).

Diverse immune cells are present in the peripheral lung parenchyma, including an abundance of alveolar macrophages. Alveolar macrophages play critical roles in innate immunity, phagocytosis and surfactant clearance in the alveoli^40,41^. In the present study MIC cells were enriched in long chained TG (Fig. 8; Tables S4 and S5) indicating increased synthesis and/or storage of these lipids. Long chained TGs were previously identified in the lungs of normal adult mice in association with increased glycerol lipases^6^. While the function of elevated long chain polyunsaturated TGs in the immune cells is unknown, emerging evidence suggests their role in lipid signaling^42,43^. Traditionally, phospholipids are thought to serve as the major source of fatty acids which upon cleavage by phospholipases, are oxidized enzymatically (e.g., lipoxygenases and cyclooxygenase) or non-enzymatically (e.g., reactive oxygen species), resulting in the formation of potent bioactive lipid mediators (LM). Recent work demonstrated that TGs are a potential source of these fatty acids^44,45^. Lysosomal acid lipase (LICH_Human), located in lysosomes, break down TGs and cholesterol esters into their associated fatty acids. In the present study LICH was selectively expressed in MIC cells in both proteomic (Figure S1) and transcriptomic (Du *et al*.^10^) studies. Lysosomal acid lipase is highly expressed in lung macrophages^46^, where it generates lipid mediators^45^. Lipid mediators have many signaling functions regulating inflammation^47^. Lipid mediators are synthesized in response to extracellular stimuli, most of which have half-lives of seconds to minutes, causing rapid and localized responses^47^. The presence of lipids enriched in fatty acids which are converted into signaling molecules within immune cells may facilitate immune signaling.

Increased alkyl-acylglycerophosphocholines (PCO) were detected in MIC cells. The PCO lipids detected in our study had the same alkyl and fatty acids chains as PCO lipids previously described in human neutrophils^48^. Abundant PCO lipids in MIC cells contained fatty acid 20:4, specifically PC(O-16:0_20:4) and PC(O-18:0_20:4) (Fig. 8 Table S5) which are known precursors of platelet activating factor (PAF; e.g., PC(O-16:0/2:0)) produced by the action of lyso-PAF acetyltransferases (LPCAT). PAF is expressed in many cell types, in particular those involved in host defense. PAF has important pro-inflammatory roles, as does the cleaved 20:4 if the fatty acid enters into the eicosanoid pathway. Although PAF was not identified in this study, proteins PCAT1_Human and PCAT2_Human (genes LPCAT1 and LPCAT2, respectively) were detected (Figure S5). PCAT1 has a well-recognized role in the synthesis of both lung surfactant and PAF. In contrast, PCAT2 is primarily associated with synthesis of PAF and is induced in inflammatory cells upon stimulation. PCAT2 was selectively expressed in proteomic (Figure S5) and transcriptomic (Du *et al*.^10^) data from MIC cell. Remaining PCO lipids may influence membrane properties, although their functions are not well defined^49^.

### Endothelial cell lipids

Endothelial cells lining the alveolar capillaries conduct blood through the lung. Their close juxtaposition to alveolar type I cells (ATI) and their permeability facilitates gas exchange. END cells maintain alveolar-capillary barrier to maintain blood flow and prevent thrombus formation^50^. Cer, PS, PEP, and PE lipids were enriched in END cells. Long chained (C35–42) PC and PI containing a 20:4 fatty acids were also increased in END cells (Tables S4 and S5). Most of these lipid classes have known roles in apoptosis and blood coagulation^51–53^ consistent with known endothelial cell functions.

Sphingolipids participate in various cellular processes including differentiation, senescence, proliferation, and signaling^15,16,51^. The balance between Cer and sphingosine-1-phosphate plays an important role in lung homeostasis; Cer causes cell cycle arrest and apoptosis^51^, while S1P facilitates proliferation and differentiation (“sphingolipid rheostat”) as well as vascular integrity^15,54^. Although Cer are enriched in the END cells, proteomics (Tables S2 and S3) and transcriptomics^10^ did not reveal enriched *de novo* ceramide synthesis, suggesting that these lipids are enriched as a part of normal lung development^15,16^ and may serve as a reservoir for routine cellular processes requiring ceramides. S1P receptor 1 (S1PR1) RNA was highly enriched in the END cells relative to other cell types^10^; consistent with the importance of S1PR1 in angiogenesis and vascular maturation^55,56^.

PS lipids serve as biomarkers for apoptosis and play a role in blood coagulation through stimulation and externalization of PS lipids from the inner to outer plasma membrane^57^. The enrichment of PS lipids in END cells in the present study was consistent with prior work that identified PS lipids in END cells^58^. PS lipids are externalized to activate prothrombinase and factor Xase promoting the coagulation cascade^11,59^ consistent with the important role END cells play in hemostasis and thrombosis^60,61^. Interestingly scramblase XKR8 RNA, which mediates exposure of PS^62,63^, was increased in END cells in transcriptomic data^10^. PE lipids, enriched in END cells in our study, may influence or play roles in endothelial membrane structure during angiogenesis^64^.

END cells react to external stimuli to regulate immune and inflammatory responses^50,65^. Long chained polyunsaturated PCs as well as PEP and PI lipids with 20:4 fatty acids were relatively increased in END cells (Table S4). These lipids serve as sources in the formation of lipid mediators, with 20:4 (arachidonic acid) acting as precursor fatty acid in prostaglandins biosynthesis, a group of lipids with multiple functions in the lung^66–68^.

### Cellular cooperation in lung function

Lipidomics of four major lung cell types (MES, EPI, MIC and END) suggested coordinated cellular networks within the human lung that support critical lung functions of gas exchange and the innate host response (see Figure S6). Previous studies support the concept that fatty acids within EPI cells are both synthesized *de novo*, recycled from the alveolar surfactant, and from circulating lipoproteins^36^. Lipidomics of MES cells support the concept that TGs present could serve as a source of fatty acids for surfactant production by AT2 cells. Our analyses highlight the role of MIC cells in degradation indicated by presence of BMP. As MIC cells are known to be active in clearing and recycling pulmonary surfactant^14^, coordination between EPI and MIC cells is likely^69^. In addition, while the metabolic pathways by which *de novo* synthesis of BMP is mediated are presently unclear, PG phospholipids which are relatively abundant in surfactant are known precursors^36,70^ making the actively recycling of PG rich surfactant an ideal source for BMPs. Of note, there is evidence that gap junctions exist that allow alveolar macrophages to communicate with other lung cells, including AT2 cells, providing a potential direct link of the metabolism of macrophages with that of surfactant producing cells^69^. Both the MIC and the END lipidome were enriched in precursor lipids that may enable the lung to mount immune responses supporting the concept that MIC and END cooperate in executing this important lung function. Interestingly, a number of these enriched lipids are dual purpose. They can both act as precursors to signaling molecules for immune response such as lipid mediators and PAF, as well as signaling molecules for apoptosis and blood coagulation; highlighting cooperative and niche cellular functions. Taken together our analyses support a posture of cellular cooperation within the human lung to support critical functions of the lung.

## Methods

### FAC Sorting

Preparation of the cells utilized is described in Bandyopadhyay, *et al.*^71^. Human lung tissue was obtained through the non-profit United Network for Organ Sharing facilitated by the International Institute for the Advancement of Medicine and the National Disease Research Interchange. Lungs were of transplant quality; researchers were offered tissue only if a suitable recipient match was not available. Consent was given for the use of tissue in research exempt from human subjects regulation due to demise yet is overseen by the University of Rochester Research Subjects Review Board protocol (RSRB00056775).

Tissue from the right upper and middle lobes were isolated and larger airway was dissected out using scissors. The remaining lung material was placed in a C-tube (Miltenyi) containing 10 ml of enzyme digestion buffer (10 mM HEPES-NaOH, 5 mM KCl, 1mMMgCl_2,_ and 1.8 mM CaCl_2_) plus a final concentration of: 2 mg/ml Collagenase Type A (Roche: 11088793001), 1 mg/ml Dispase II (Gibco: 17105-041), 0.5 mg/ml (1.5 units/mL) Elastase (Worthington: ESL), and 2 mg/ml (800 units/ml) Deoxyribonuclease-I (Sigma: DN-25). Tissue was minced with scissors and the C-tube was placed on the GentleMACS system (Miltenyi). Tissue disruption was completed using the mouse tumor 01.01 program. The C-tube was placed, with the cap loosened, at 37 °C in 5% CO_2_ for 1 hour, while inverting every 15 minutes. Digested material was passed through a 100 micron strainer into a 50 ml conical tube using the plunger of a syringe. The conical tube was then centrifuged (1000xg at 4 °C for 10 min) and supernatant was decanted. A red blood cell lysis step was performed by resuspending cells in 10 ml of ACK solution (Biowhittaker: Cat#10–548E). ACK was neutralized using 30 ml of 10% FBS in PBS and cells were pelleted by centrifugation (800 × g at 4 °C for 10 min). Cells were then cryopreserved in a solution of 90% FBS + 10% DMSO and frozen to −80 °C at a slow rate using the Mr. Frosty system (Nalgene). Cells were placed in a liquid nitrogen vapor phase cryopreservation unit for long term storage.

Cells were thawed quickly and transferred into a 15 ml conical tube. To wash cells, 10 ml of PBS + 10% FBS was added dropwise, followed by centrifugation (800 × g at 4 °C for 10 min). Supernatant was removed and cells were resuspended in PBS/FBS, mixed with trypan blue viability dye and then counted by hemacytometer. In preparation for sorts, cellular staining was performed at a final dilution of 10 µl staining cocktail per 1 million cells. Briefly, cells were resuspended in a 4% normal mouse serum (Sigma: M5905) solution in PBS + 2% BSA (5 µl per 1 million cells) and incubated on ice for 10 minutes. A staining cocktail consisting of 1:50 Podoplanin-AF647 (Biolegend: 337008), 1:50 CD31-BV605 (BD: 562855), 1:50 CD326-PE (eBiosceince: 12-9326-42), 1:50 CD45-V450 (BD: 560367), CD144-FITC (BD: 560411), and 1:800 CD235a (BD: 559944) was added to cells (5 ul per 1 million cells) for 80 min on ice in the dark. Cells were washed, filtered, and sorts were completed using a FACSAria II (BD) instrument. Cell fractions were: CD45+ mixed immune cells (MIC), CD31/144+ endothelial (END), CD326+ epithelial (EPI), and stained but not sorted mixed cells (PMX) (Fig. 1). Cells were collected by centrifugation (1000 × g at 4 °C for 10 min). The pellet was resuspended in 1 ml of PBS and transferred to 1.7-ml Sorenson Bioscience MulTI SafeSeal Microcentrifuge (VWR), and re-pelleted (2000 × g at 4 °C for 10 min). Supernatant was removed and cells were snap frozen for downstream proteomic and lipidomic analysis.

### Lipid and protein extraction

Lipids and proteins were generated using a modified Folch extraction^12^ enabling multi-omics analysis^72^. Briefly, cells were lysed adding 300 µl of methanol to the samples then they were sonicated for 1 min and incubated in an ice bath for 1 min. Sonication and the ice bath were repeated 2 more times. Samples were then transferred into vials containing 600 µl of chloroform, vortexed, and 225 µl of water was added to allow for a phase separation. The samples were vortex gently to mix, incubated for 5 min in an ice chilled sample holder, vortexed for 10 s and then centrifuged at 10,000 x g for 10 min. The total lipid extract (TLE) was transferred into a glass vial, dried in a speedvac, and then reconstituted in 500 µl 1:1 chloroform/methanol for storage at −20 °C until analysis. The protein pellet was also removed, dried in a speedvac, then reconstituted in 30 µl of 8 M urea containing 50 mM of ammonium bicarbonate and underwent tryptic digest as outlined below.

### Protein Digestion

The protein extracts were reduced with DTT (5 mM for 30 min at 60 °C), then alkylated with iodoacetamide (400 mM for 1 h at 37 °C in the dark), diluted 10 times in 50 mM ammonium bicarbonate containing 1 mM of CaCl_2_ prior to digestion. Resulting peptides were desalted using C18 SPE cartridges (Discovery C18, 1 mL, 50 mg, Sulpelco). The peptide concentrations were measured by BCA assay (Thermo Scientific).

### Mass spectrometry analysis and molecular identifications

Samples were analyzed using liquid chromatography tandem mass spectrometry (LC-MS/MS). Lipids were analyzed and identified as outlined in Kyle *et al*.^21^. Briefly, TLEs were dried in vacuo and reconstituted in 50 µl methanol, 10 µl of which was injected onto a reversed phase Waters CSH column (3.0 mm × 150 mm × 1.7 µm particle size) connected to a Waters Aquity UPLC H class system interfaced with a Velos-ETD Orbitrap mass spectrometer. Lipid molecular species were separated over a 34 min gradient (mobile phase A: ACN/H_2_O (40:60) containing 10 mM ammonium acetate; mobile phase B: ACN/IPA (10:90) containing 10 mM ammonium acetate) at a flow rate of 250 µl/min. Samples were analyzed in both positive and negative ionization using HCD (higher-energy collision dissociation) and CID (collision-induced dissociation) to obtain high coverage of the lipidome. Confident lipid identifications were made using in-house developed identification software LIQUID (Kyle *et al*.^21^) where the tandem mass spectra was examined for diagnostic ion fragments along with associated hydrocarbon chain fragment information. To facilitate quantification of lipids, a reference database for lipids identified from the MS/MS data was created and features from each analysis were then aligned to the reference database based on their identification, *m/z* and retention time using MZmine 2 (Pluskal *et al*. 2010). Aligned features were manually verified and peak apex intensity values were exported for subsequent statistical analysis.

An Agilent 6560 IM-QTOF MS (Agilent Technologies, Santa Clara) was used for the IMS-MS measurements^73–75^. The Agilent 6560 was outfitted with a commercial gas kit (Alternate Gas Kit, Agilent) and a precision flow controller (640B, MKS Instruments) to allow for real-time pressure adjustment based on the drift tube pressure using a capacitance manometer (CDG 500, Agilent). For the DTIMS measurements, ions were passed through an inlet glass capillary, focused by a high-pressure ion funnel, and accumulated in an ion funnel trap. Ions were then pulsed into the drift tube filled with ~3.95 torr of nitrogen gas, where they travelled under the influence of a weak electric field (10–20 V/cm). Ions exiting the drift tube were refocused by a rear ion funnel prior to QTOF MS detection and their arrival times were recorded. IMS-MS data were collected from 50–1700 *m/z* with a cycle time of 1 sec/spectra to increase the signal of low abundance species. The PG and BMP lipid standards were purchased from Avanti Polar Lipids, Inc. Prior to LC-IMS-MS analysis, the standards were diluted to 0.001 µg/µl with 100% methanol.

For proteomics analysis, 5 μl of 0.1 μg/μl of peptides were analyzed by reverse phase separation (C18) using a Waters nanoEquityTM UPLC system interfaced with a QExactive Plus Orbitrap mass spectrometer. Briefly, peptide samples were first loaded on a solid phase extraction (SPE) column (5 cm long × 150 um ID, Jupiter C18, 3 µm particles) via a 5 µl sample loop for 30 min at a flow rate of 3 µl per minute using mobile phase A and then separated over a 180 min gradient on an analytical column made in-house by slurry packing 3-μm Jupiter C18 stationary phase into a 70-cm long, 360 μm OD × 75 μm ID fused silica capillary tubing (Mobile phase A: 0.1% formic acid in water; mobile phase B: 0.1% formic acid in acetonitrile) at a flow rate of 0.3 µl/min. Mass spectrometry analysis was initiated 15 minutes after the separation gradient started. The LC effluent was ionized by electrospray ionization in positive ionization mode by applying 2200 volts on the metal union between the column and the spray tip and resulting ions were transferred into the mass spectrometer using a 360 um ID capillary heated at 300 degree C. A primary survey scan was made in the mass range of m/z 300 to 1800 at a resolution of 35 k, automated gain control (AGC) setting of 3E6 and ion injection time of 20 ms. From this scan, top 12 ions were selected by a quadrupole mass filter using isolation width of 2 m/z for high energy collision dissociation (HCD) at a normalized collision energy of 30% and resulting fragment ions were mass analyzed by the Orbitrap at a resolution of 17500, AGC setting at 1E5 and maximum injection time of 100 ms. Mass spectra were recorded for 180 minutes by repeating this process with a dynamic exclusion of previously selected ions for 30 seconds. Identification and quantification of the proteins was performed using MaxQuant software as previously described^5^.

### Statistics

The abundance values were log2 transformed and median normalized for both the proteins and the lipids. For the proteins, the missing values were imputed by the minimum value of the resulting table divided by two. For the lipids, the species containing missing data were discarded for quantification. The Student tests, the ANOVA, the unsupervised hierarchical clustering were performed using the stat package from R (Version 3.4.0). The PCA were realized using the FactoMineR package^76^. GO enrichments were performed using DAVID bioinformatics resources^20^. The figures were generated in R using the package ‘plot3D’, ‘gplot’ and ‘ggplot2’ or Microsoft Excel 2010 and visually adjusted in Adobe Illustrator (version 16.0.5).

### Accession Numbers

Data deposited and freely available at MassIVE data repository, MassIVE ID: MSV000081973.

## Electronic supplementary material

Supplemental Information

Dataset
