# Supplementary material for: Cell type-resolved human lung lipidome reveals cellular cooperation in lung function
Source: Sci Rep. 2018 Sep 7;8:13455. doi: 10.1038/s41598-018-31640-x (PMC6128932; doi:10.1038/s41598-018-31640-x)
Supplement: Supplementary file 1 — Supplemental Information [file 41598_2018_31640_MOESM1_ESM.pdf]

Cell type-resolved human lung lipidome reveals cellular cooperation in lung function

Jennifer E. Kyle[1], Geremy C. Clair[1], Gautam Bandyopadhyay[3], Ravi S. Misra[3], Erika M. Zink[1], Kent J. Bloodsworth[1], Anil K. Shukla[1], Yina Du[2], Jacquelyn Lillis[4], Jason R Myers[4], John Ashton[4], Timothy Bushnell[5], Matthew Cochran[5], Gail Deutsch[7], Erin S. Baker[1], James P. Carson[6], Thomas J. Mariani[3], Yan Xu[2], Jeffrey A. Whitsett[2], Gloria Pryhuber[3], Charles Ansong[1*]

[1]Biological Sciences Division, Pacific Northwest National Laboratory, Richland, WA, 99352, USA
[2]Division of Pulmonary Biology, Cincinnati Children's Hospital Medical Center, Cincinnati, OH 45229, USA
[3]Department of Pediatrics, [4]UR Genomics Research Center, [5]UR Flow Cytometry Core Facility, University of Rochester School of Medicine and Dentistry, 601 Elmwood Avenue, Rochester, NY 14642, USA
[6]Texas Advanced Computing Center, University of Texas at Austin, Austin, TX 78712, USA
[7] Department of Pathology, Seattle Children's Hospital, Seattle, WA 98105, USA

*Corresponding author: Charles Ansong

Address: 902 Battelle Blvd.
P.O. Box 999, MSIN K8-98
Richland, WA 99352
Phone: 509-371-6327
Email: charles.ansong@pnnl.gov

Keywords
Lipidomics, proteomics, mass spectrometry, lung development

Supplemental Figure Legends.

Figure S1. Average lysosomal acid lipase abundance expressed in the proteome (LICH_Human) in the four cell populations across the three donors.

Figure S2. Average abundance of lipoprotein lipase (LPL) and perilipin-2 (PLIN2) transcripts in the four cell populations across the three donors. Cognate proteins not detected in proteome dataset.

Figure S3. Heatmap of PC lipids elevated in the EPI cells. Data in the heatmap is z-scored. SumC represents the total number of carbons in the fatty acids chains, and #DB represents the total number of double bonds in the fatty acids chains. The p-values highlighted red are statistically significant (p-value ≤ 0.05) and in red bold text for those with p-values ≤ 0.01. Note the elevated PC lipids have low sumC and #DB.

Figure S4. Average abundance of surfactant proteins in the four cell populations across the three donors. Cognate transcripts not shown.

Figure S5. Average abundance of PCAT1 protein (A), PCY1A protein (B) and PCAT2 protein (C) in the four cell populations across the three donors.

Figure S6. A model of cellular cooperation in lung function. Lipidomics and proteomics analysis of cell-resolved human lung tissue revealed mesenchymal, epithelial, and immune cells have interconnected functions in surfactant metabolism. Additionally immune and endothelial cells have interconnected roles related to lipid-signaling and immune response. We thank Ms. Rose Perry for graphic art assistance in drawing images in Figure S6.

Figure S1

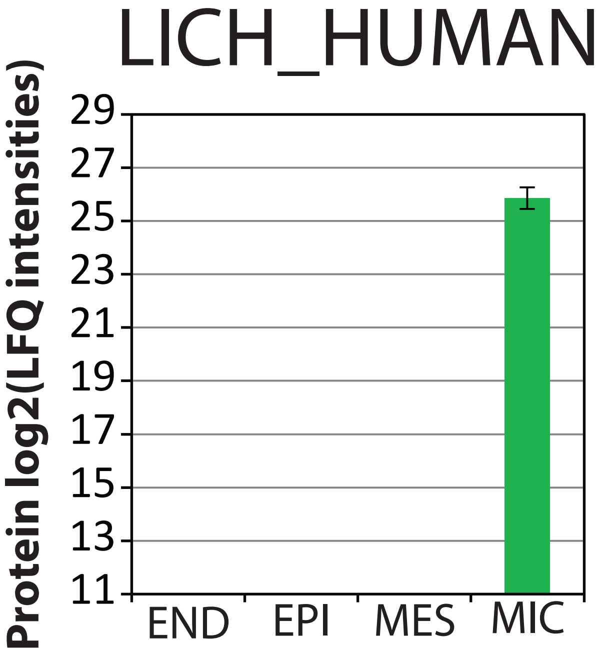

LICH_HUMAN
Protein log2(LFQ intensities)
29
27
25
23
21
19
17
15
13
11
END
EPI
MES
MIC

Figure S2

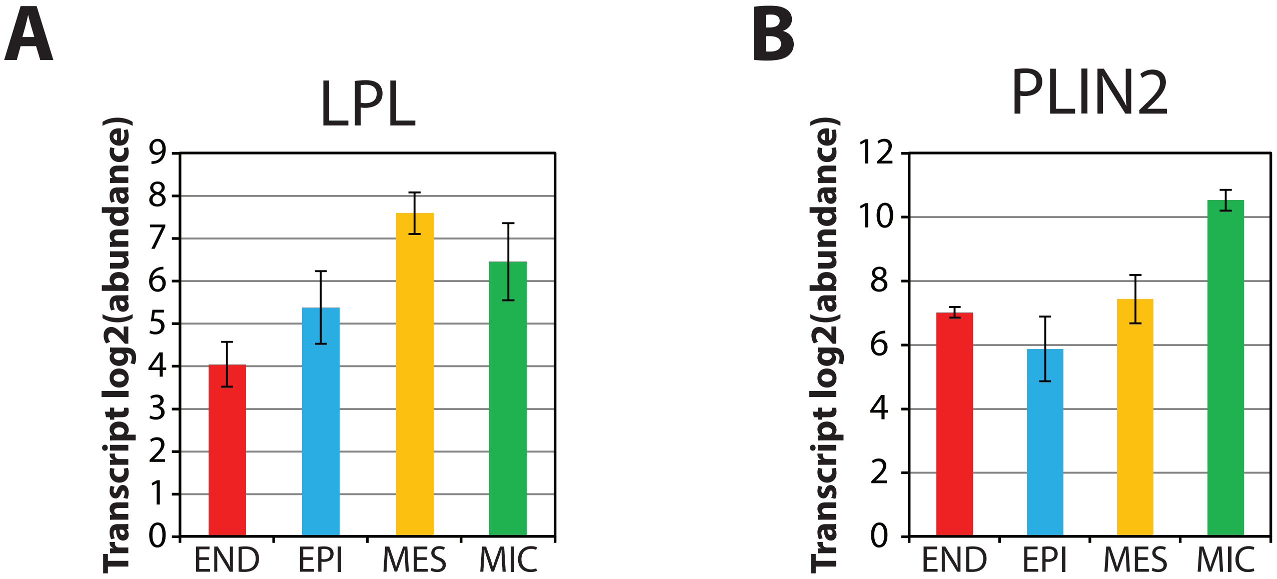
A
B
LPL
PLIN2
Transcript log2(abundance)
Transcript log2(abundance)
0
1
2
3
4
5
6
7
8
9
0
2
4
6
8
10
12
END
EPI
MES
MIC
END
EPI
MES
MIC

Figure S3

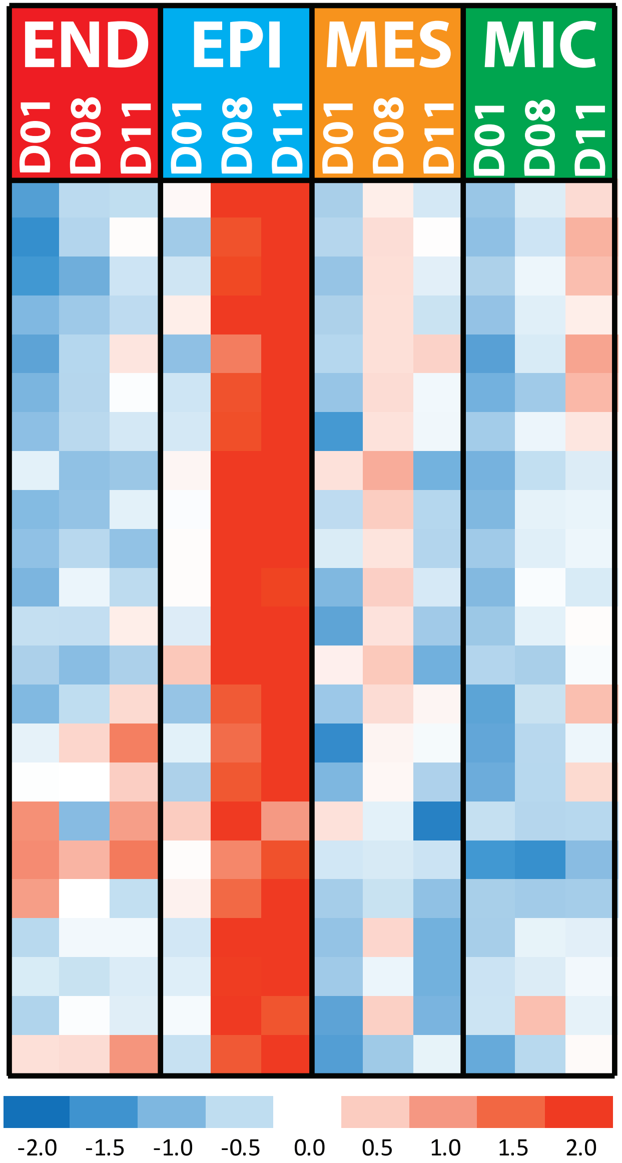

Lipid	SumC	#DB	pvalue
PC(14:0/14:0)	28	0	0.028
PC(14:0/15:0)	29	0	0.294
PC(14:0/16:0)	30	0	0.076
PC(14:0/16:1)	30	1	0.020
PC(14:0/17:0);PC(15:0/16:0)_A	31	0	0.566
PC(15:0/16:0)_B	31	0	0.170
PC(15:0/16:1)	31	1	0.122
PC(16:0/16:0)	32	0	0.034
PC(14:0/18:1);PC(16:0/16:1)_A	32	1	0.033
PC(16:0/16:1)_B	32	1	0.016
PC(14:0/18:2)_A	32	2	0.041
PC(14:0/18:2)_B;PC(16:1/16:1)	32	2	0.100
PC(16:2/16:0)	32	2	**0.006**
PC(16:0/17:0)	33	0	0.421
PC(15:0/18:1);PC(16:0/17:1)	33	1	0.116
PC(15:0/18:2)	33	2	0.178
PC(16:0/18:0)	34	0	0.102
PC(16:0/18:1)	34	1	**0.000**
PC(16:0/18:2)	34	2	0.018
PC(16:1/18:1)	34	2	0.089
PC(16:0/18:3)	34	3	0.036
PC(16:1/18:2)	34	3	0.099
PC(14:0/20:4)	34	4	0.060

Figure S4

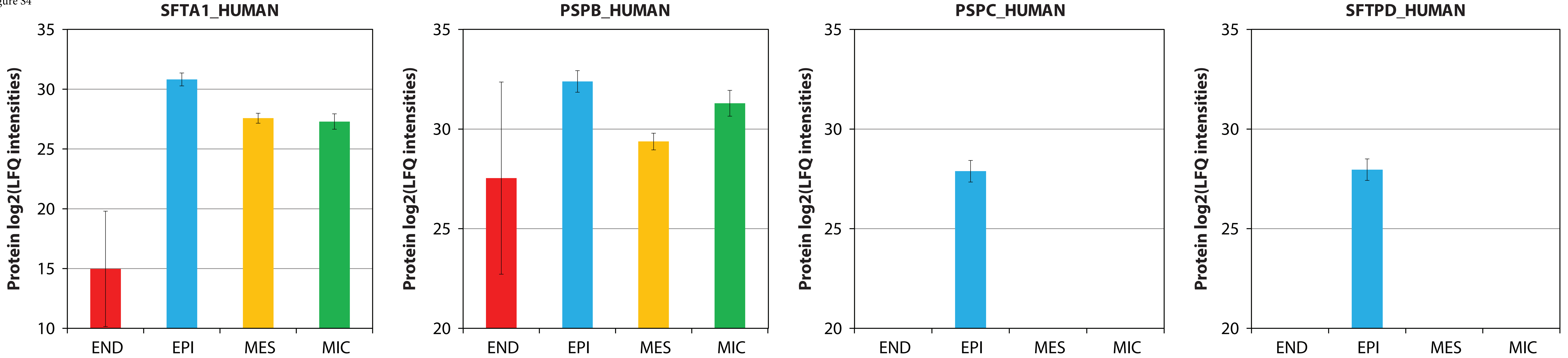
SFTA1_HUMAN
PSPB_HUMAN
PSPC_HUMAN
SFTPD_HUMAN
Protein log2(LFQ intensities)
35
30
25
20
15
10
END
EPI
MES
MIC

Figure S5

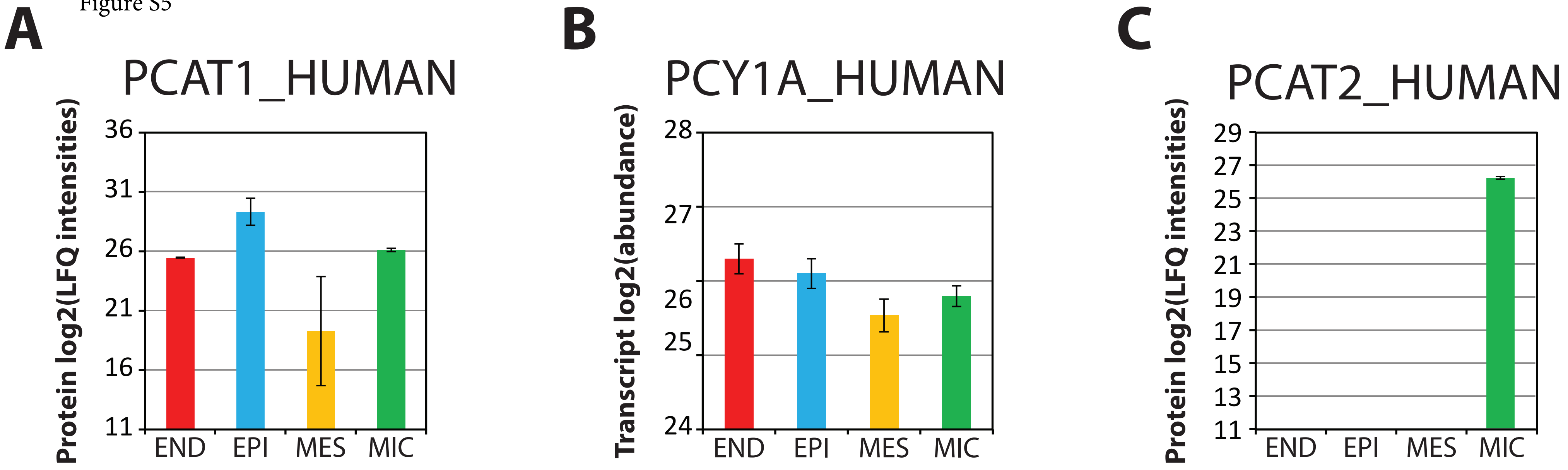
A
PCAT1_HUMAN
Protein log2(LFQ intensities)
36
31
26
21
16
11
END
EPI
MES
MIC
B
PCY1A_HUMAN
Transcript log2(abundance)
28
27
26
25
24
END
EPI
MES
MIC
C
PCAT2_HUMAN
Protein log2(LFQ intensities)
29
27
25
23
21
19
17
15
13
11
END
EPI
MES
MIC

Figure S6

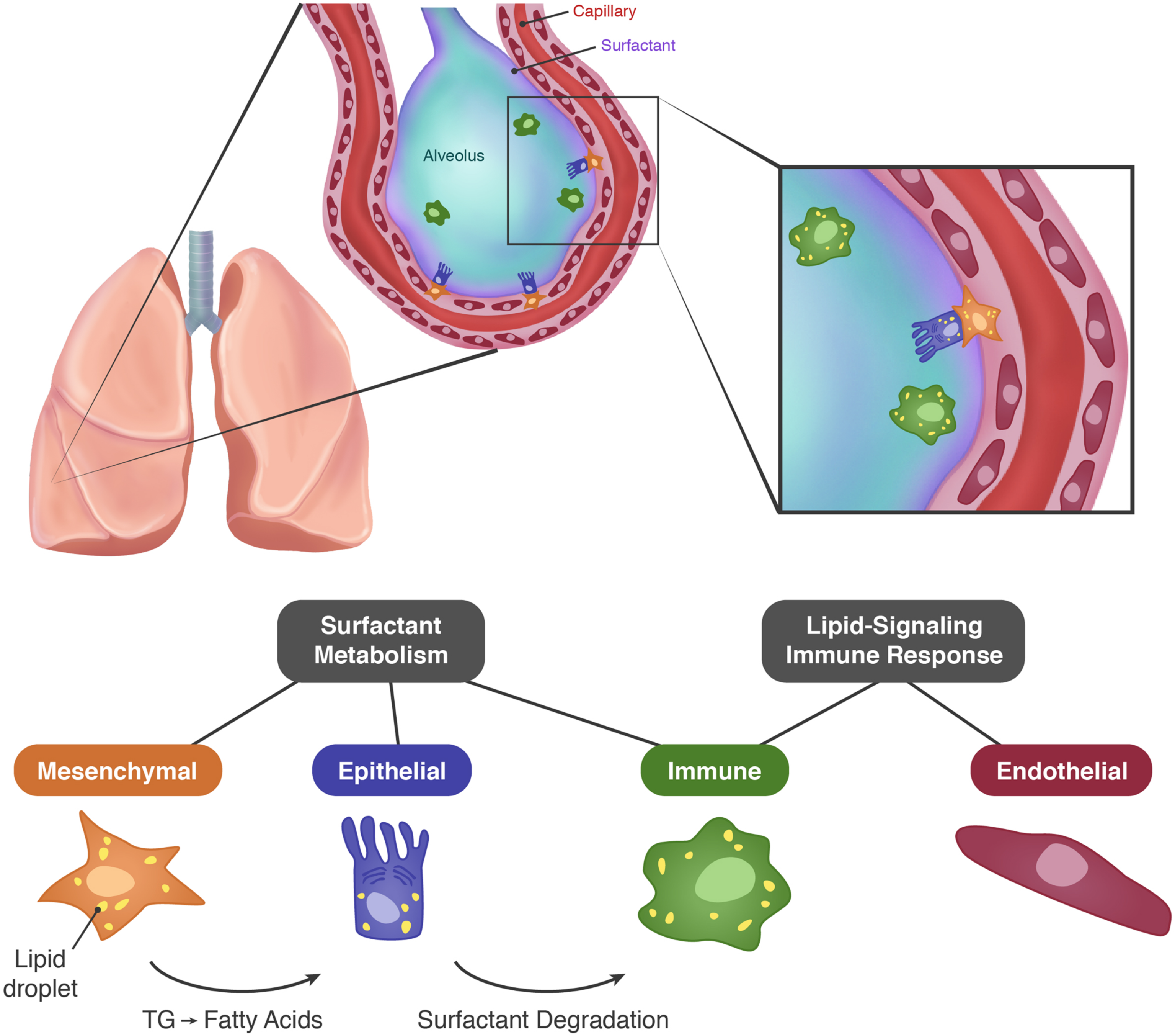

Capillary
Surfactant
Alveolus
Surfactant Metabolism
Lipid-Signaling Immune Response
Mesenchymal
Epithelial
Immune
Endothelial
Lipid droplet
TG → Fatty Acids
Surfactant Degradation
